# Use of Natural Antioxidants from Newfoundland Wild Berries to Improve the Shelf Life of Natural Herbal Soaps

**DOI:** 10.3390/antiox8110536

**Published:** 2019-11-08

**Authors:** Oludoyin Adigun, Charles Manful, Natalia Prieto Vidal, Abira Mumtaz, Thu Huong Pham, Peter Stewart, Muhammad Nadeem, Dwayne Keough, Raymond Thomas

**Affiliations:** 1 School of Science and the Environment/Boreal Ecosystem Research Initiative, Grenfell Campus, Memorial University of Newfoundland, 20 University Drive, Corner Brook, NL A2H 5G4, Canada; cfmanful@mun.ca (C.M.); nprietovidal@grenfell.mun.ca (N.P.V.); abiramumtaz@gmail.com (A.M.); tpham@grenfell.mun.ca (T.H.P.); pstewart@grenfell.mun.ca (P.S.); mnadeem@grenfell.mun.ca (M.N.); 2 School of Life Sciences and Environment, Algoma University 1520 Queen St E., Sault Ste. Marie, ON P6A 2G4, Canada

**Keywords:** natural herbal soap, antioxidant, unsaponified neutral lipids, shelf life

## Abstract

Antioxidants are important bio-regulators and suppressors of oxidation and are useful in enhancing the shelf life of consumer products. Formulated natural herbal soaps contain ingredients with antioxidant activities, but it is unknown how this influences shelf life. Herein, we evaluated whether natural additives or wild berry extracts were effective in improving the quality of natural herbal soaps. Three natural soaps, base bar (BB), forest grove (FG), and hibiscus rosehip (HR), were formulated using several wild berry extracts or natural additives and evaluated against similar commercial brands. The total phenolic content (TPC) of BB and FG infused with partridgeberry and HR with rosemary was 35.22, 44.72, and 33.26 µmole quercetin equivalent/g soap, while the total antioxidant activity (TAA) was 125.20, 119.23, and 126.94 µmole Trolox equivalent/g soap, respectively. Conversely, the commercial brand (BSG) with the highest TPC (56.24 µmole) contained lower TAA (59.68 µmole). As expected, the TPC and TAA of natural soaps were strongly correlated, and the majority (55–82%) of the polyunsaturated di/triacylglycerols remained unsaponified. Some extracts were inhibitory, while others promoted microbial growth. The results indicate that natural antioxidants from some Newfoundland wild berries have applications in improving the shelf life of natural herbal soaps, but care must be taken with the choice of berry used in the final soap formulation.

## 1. Introduction

The soap industry is one of the oldest chemical industries that provides cleaning agents for the personal skincare, restaurant, food, and laundry sectors [1]. Soap was initially produced thousands of years ago by reacting animal fats and ashes obtained from plants [2]. In the early years, soap was hardly used for personal hygiene but mainly for laundry. The unpleasant characteristics of the finished products, such as offensive odors, excessive amounts of unreacted caustic soda, and unsaponified lipids, determined the quality of the final product thus limiting its use in personal hygiene. 

Currently, soaps are produced via processing methods, such as kettle boiling, hydrolysis/neutralization, continuous or cold saponification using different fats and feedstocks, yielding final products having characteristics more suitable for applications in personal hygiene [3,4]. The current status of economic growth in the soap industry as well as trends in consumer preferences have led to increased demands for the use of natural ingredients as additives in personal skin and cosmetic products [5]. As such, natural herbal soaps formulated using different oils and plant-based additives are a major segment and have high demand in the global market. 

Soaps are manufactured via saponification reaction where triacylglycerols or free fatty acids used in the feedstock are reacted with an alkali to produce metal salts [3]. Triacylglycerols (TGs) are the major components of vegetable oils and animal fats that are reacted with sodium hydroxide (or other mineral bases) in a aqueous medium to produce metal salts of hydrolyzed free fatty acids (soaps) and glycerol as a byproduct [3]. Diacylglycerols (DGs) and monoacylglycerols (MGs) can also be present in the final product, unsaponified, due to the fact of incomplete hydrolysis, and they are important components modulating the final characteristics of the soap produced. 

Cold saponification is a technique preferred by craft producers during natural soap production, because it allows more flexibility in the choice of feedstock materials, superior retention of natural antioxidants, color, and essential oil fragrance in the final product [3]. Furthermore, the sensory attributes and chemical properties of natural soaps also depend on the method used for manufacturing. For instance, the type of strong mineral base (alkali) used determines the hardness and solubility of the final soap product. Hard soaps are produced from oils such as those obtained from palm kernel containing high-saturated fatty acids and saponified with sodium hydroxides (also known as lye). Conversely, soft soap bars (semi-liquid) or liquid soaps are made from oils such as cottonseed oil with high levels of unsaturated fatty acids typically saponified with potassium hydroxide [6,7,8]. 

The formulations and chemical composition determine soap classification which could be toilet, antiseptic, or medicated soaps. Antiseptic and medicated soaps are produced to fight pathogenic microbes due to the inclusion of chemical additives in the formulation, while toilet soaps are produced mainly for conventional cleansing purposes. However, some consumers have sensitivity to chemical additives used to improve the soap organoleptic quality [9]. This has led to increased demands from consumers for the use of natural additives in soaps as well as a steady growth in the artisanal natural soap sector of the cosmetic industry to provide specialty hand-crafted herbal soaps customized with various natural ingredients as an alternative for consumers or a product more suited for consumers with skin sensitivities [4]. 

Approximately 70–85% of the total contents of natural herbal soaps are made up of sodium salts of saponified fatty acids derived from triglycerides or hydrolyzed fatty acids present in the feedstocks [3,5]. These fatty acids play active roles in the natural soap performance, consumer preference, and the price of the product. The length of the carbon chain, degree of unsaturation (number of double bonds), and the composition and distribution of the saponified lipids are major determinants of the finished soap performance. Saturated lipids provide light open foams (lather) and a solid and hard consistency, while unsaturated fatty acids provide moisturizing, conditioning, or skin nourishing properties to the final soap product [3,6,10,11,12,13,14]. 

Mixtures of coconut, olive, palm, rice bran, and sunflower seed oils are major sources of oils commonly used in natural herbal soaps. In some cases, animal fat and vegetable oil may be mixed in the final formulations to modify the performance of the finished product. However, vegetable oils are mostly used because they produce higher quality soaps rich in polyunsaturated lipids derived from glycerolipids (MGs, DGs, and TGs) [3,10,12,13]. Vegetable oils such as soybean or olive oils contain significant levels of monounsaturated, polyunsaturated, and long-chain saturated fatty acids as major constituents. 

The unsaturated fatty acids in the oils used as soap feedstock are highly susceptible to oxidation. Chemical compounds (antioxidants) such as butylated hydroxytoluene (BHT) and butylated hydroxyanisole (BHA) are commonly used as additives to suppress lipid oxidation in oils used as feed stock during soap production. However, consumer demand for natural antioxidants combined with safety and health implications associated with synthetic antioxidants have resulted in increased use of natural antioxidants in soaps, skin care, and cosmetic products [15]. Certain regulatory factors must be considered during the formulation of cosmetic products such as the concentration of the recipes required to enhance the quality and shelf life of the final product. In this regard, the inclusion of active and effective natural antioxidants is very important. In the past decade, some natural plant extracts were found to possess broad spectrum inhibitory effects on different types of pathogenic organisms such bacteria, fungi, yeast, and molds known to affect product quality and shelf life. Wild berry extracts are found to contain high levels of phenolic compounds which have been demonstrated to have antimicrobial activity in in vitro and in vivo studies [16,17]. These extracts were observed to contain a variety of secondary metabolites, such hydroxycinnamic acid, alkaloids, flavonoids, and phenolics, with antimicrobial properties [4,18,19]. In particular, phenolics are gaining more attention in the cosmetic industry due to the fact of their dual functional role as antioxidants and antimicrobials [4,20]. Therefore, the use of phenolics as additives in natural herbal soap formulations to prevent the oxidation of polyunsaturated lipids and enhance the shelf life is becoming more common place with artisanal soap makers [18]. These include plant, fruit, and vegetable extracts [14,15]. Furthermore, plant extracts are also used as colorants and to add fragrance to natural herbal soaps [21]. 

Many artisanal soap producers employ cold saponification along with combinations of different vegetable oils, essential oils, and plant extracts to produce various herbal soaps [3]. However, to the best of our knowledge, there is a paucity of information in the scientific literature concerning how antioxidants from different botanical sources may be effective in enhancing the shelf life and overall quality of natural herbal soaps. Therefore, the aim of this present study was to understand the effects of antioxidants from Newfoundland wild berries on natural herbal soap shelf life in terms of preservation against lipid oxidation and microbial growth. We hypothesized that the shelf life and level and composition of unsaponified lipids present in natural herbal soaps will be significantly affected by the composition of the feedstocks and additives used as antioxidants to produce natural herbal soaps following cold saponification, and that careful considerations should be given to the choice of extracts used by artisanal soap makers during formulation and production.

## 2. Materials and Methods 

### 2.1. Soap Production by Cold Saponification 

Commercial natural herbal soaps were manufactured in collaboration with the industry using 6% super fat (lye discount) and 35% water. A commercial lye calculator was used to calculate the rate of saponification for each oil used to formulate each soap. The soaps were manufactured by a cold saponification or cold processing method. Sodium hydroxide (NaOH) or lye was used as the base. The aqueous solution of NaOH_(aq)_ was prepared and then allowed to cool for 60 min. A mixture of oils, including castor oil (5%), palm oil (20%), soybean oil (20%), olive oil (20%), coconut oil (30%), and butter (5%), was added to the NaOH_(aq)_ solution and properly blended to homogenously mix the ingredients until just before trace was achieved. Parchment paper-lined molds were filled with the soap mixture and left to saponify at room temperature for 24 h. Following completion of the saponification process, each loaf of soap was removed from the mold, weighed (1.5 kg approximately), and the dimensions measured (0.05 × 0.08 × 0.38 m). The loaves were cut into 15 identical bars simultaneously using a commercial wire loaf cutter. Each bar had an equal shape, with dimensions of 0.03 × 0.05 × 0.07 m and weighed approximately 0.1 kg. The bars were loaded into plastic containers and allowed to cure for a month by storing at room temperature in a dark room. After curing, 0.008 kg soaps were cut from the center of each soap using a surgical blade wrapped with aluminum foil paper and kept in −80 °C for further analyses. The following abbreviations were used to delineate the natural herbal soaps evaluated in this study: BB = base bar, FG = forest grove, HR = hibiscus rosehip, A = essential oil blend additives, L = blueberry, P = partridge berry, S = rosehip, C = cranberry, M = rosemary extracts. 

### 2.2. Addition of Natural Additives to Herbal Soaps 

To determine the effects of natural additives on the shelf life of natural herbal soap produced following cold saponification, natural plant additives from rosemary, rosehip, blueberry, and partridge berry were added to each of the soaps produced as follows: 750 mg of berry extract was added to a loaf of soap based on the Food and Drug Administration’s (FDA) guidelines, i.e., 0.02–0.05% of finished product weight. Additional details on soap formulation and manufacturing are as described by our previous publication [3].

### 2.3. Chemical Analysis of the Natural Herbal Soaps

#### 2.3.1. Extraction of Samples

Extraction of samples was carried out according to the methods of Thomas et al. [13] and Cano et al. [22] with minor modifications suitable to obtain the hydrophilic and lipophilic phenolic content, antioxidant activities, and oxidation status of each soap. Samples (100 mg each) from the four different batches of soaps were weighed in four (4) replicates into glass centrifuge vials. The small pieces of the soaps were cut and placed into 1 mL of HPLC grade acetone:ethanol (1:1 *v/v*) to form a solution. The soap solutions were centrifuged at 10,000× *g* for 15 min, and the supernatant was carefully decanted without disturbing the pellet. The supernatant was filtered using glass wool, and the filtrate was used directly without extra dilution to determine the lipophilic antioxidant activity and the organic phenolic content of the soaps. To the undisturbed pellets, 1 mL of cold 50 Mm sodium phosphate buffer (pH 7.5) was added, and centrifuged for 15 min at 10,000× *g*, and the supernatant was carefully decanted. The decanted supernatant was further diluted 1:10 with 50 Mm sodium phosphate buffer (pH 7.5) and then used for the determination of aqueous antioxidant activity and hydrophilic phenolic content.

#### 2.3.2. Total Phenolic Content (TPC) Analysis

The hydrophilic and lipophilic phenolic content of the soaps were measured separately using a 10 fold diluted solution of Folin-Ciocalteu reagent with quercetin as a standard in the range of 0–1.0 mg/mL [13,19]. The aqueous phenolic (hydrophilic) and organic phenolic (lipophilic) were determined by mixing a 5 µL extract of the samples with the buffer or organic solvent, respectively. To the sample mixture, 130 µL of Folin-Ciocalteu reagent and 75 µL of either ethanol:acetone (1:1 *v/v*) or 50 Mm sodium phosphate buffer (pH 7.5) were added to microplate wells for the determination of lipophilic and hydrophilic phenolic content, respectively. The resultant mixtures in microplates were incubated for 30 min in the dark at room temperature, and the absorbance measured at 755 nm using a Synergy HT microplate reader (Biotek, Fisher Scientific, Mississauga, ON, Canada). The units of the results are expressed as micromole quercetin equivalents per gram of soap. Four replications per standard concentration or sample treatment were analyzed. The value of total phenolic content was determined by the addition of the hydrophilic and lipophilic phenolic values.

#### 2.3.3. Antioxidant Activity Analysis (FRAP Method)

Determination of hydrophilic and lipophilic antioxidant activities were carried out according to the methods of Jimenez-Alvarez et al. [23]. This protocol followed the capacity of a sample to scavenge the FRAP radical cation compared to a standard antioxidant (Trolox) in a dose–response curve (0–100 µM). The fresh reaction mixture was prepared daily by adding solutions of 25 mL of 30 mM acetate buffer (pH 3.6), 2.5 mL of 10 mM tripyridyltriazine (TPTZ), and 2.5 mL of 20 mM FeCl_3_·6H_2_O to form a working solution. This working solution was heated to 37 °C in an oven for 15 min before use. A standard solution of 1 mM of Trolox was produced by dissolving 6.25 mg Trolox in 25 mL of sodium phosphate buffer for the hydrophilic antioxidant activity or acetone:ethanol (1:1 *v/v*) for the lipophilic antioxidant activity. For determination of hydrophilic and lipophilic antioxidant activities, 10 µL of sample or standard was added to microplate wells containing 190 µL working solution. The resultants mixtures were incubated for 30 min in the dark and the absorbance measured at 593 nm using a Synergy HT microplate reader (Biotek, Fisher Scientific, Mississauga, ON, Canada). Four replications per standard concentration or sample treatment were analyzed. The units of the results are expressed as micromole Trolox equivalents per gram soap. The total antioxidant activity was determined by addition of the hydrophilic and lipophilic antioxidant values.

#### 2.3.4. Total Oxidant Status (TOS) Analysis

The established method of Erel [24] was used for the total oxidant status (TOS) assay. The method is based on the oxidation of ferrous ions and an o-dianisidine complex in the assay to form ferric ions by oxidants present in the soap samples. The ferric ions form a chromophore with xylenol orange in an acidic medium, and the amounts of oxidant molecules present in the samples can be determined via a linear relationship with known standards by measuring absorbance at 560 nm. Reagent 1 stock solution was made up of 114 mg of xylenol orange, 100 mL of glycerol, 8.18 g of sodium chloride, and 900 mL of 25 mM H_2_SO_4_ (1.2 mL of 98% H_2_SO_4_ (d = 1.84 g/L) in 900 mL H_2_O) to give a final composition of 150 µM xylenol orange, 140 mM NaCl, 1.35 mL glycerol, and a pH of 1.75. Reagent 2 stock solution was prepared by dissolving 1.96 g of ferrous ammonium sulfate and 3.17 g o-dianisidine dihydrochloride in 1000 mL of 25 mM H_2_SO_4_ (1.33 mL of 98% H_2_SO_4_ (d = 1.84 g/L) in 1000 mL of H_2_O). The composition of this reagent was 5 mM ferrous ammonium sulfate and 10 mM o-dianisidine dihydrochloride. A standard solution of 1 mM hydrogen peroxide in water was serial diluted to prepare working calibration standard solutions of 0, 25, 50, 100, and 150 µM. For this assay, the mixture of 35 µL of sample or standard, 225 µL of Reagent 1, and 10.6 µL of Reagent 2 was placed in microplate wells. The absorbance was measured at 560 nm using a Biotek Synergy HT microplate reader (Fisher Scientific, Mississauga, ON, Canada). Four replicates per standard concentration or sample treatment were analyzed for the determination of lipophilic and hydrophilic oxidant status. The units of the results are expressed as µM equivalents of H_2_O_2_ per gram of soap. The value of total oxidant status was determined by addition of the hydrophilic and lipophilic oxidant values.

#### 2.3.5. Soap Lipid Extraction

Extraction of lipids from soaps was carried out by weighing 50 mg of soap sample into 2 mL glass vials in four replicates, and extraction was done following the established Bligh and Dyer method with the following modifications [3,23,25]. Each soap sample was suspended in 1 mL of methanol containing 0.01% butylated hydroxytoluene (BHT), and the sample mixture was thoroughly vortexed. Then, 0.8 mL distilled water and 1 mL chloroform were added, and the mixture was centrifuged at 10,000× *g* for 10 min. The organic layer was retained for determination of unsaponified lipids in the soaps.

#### 2.3.6. Lipid Analysis

The method used for lipid analysis is described in a previous publication by our group [26]. Briefly, unsaponified complex lipids (oils) extracted from natural soap were separated using an Accucore C30 column (150 × 2 mm I.D., particle size: 2.6 µm, pore diameter: 150 Å obtained from ThermoFisher Scientific, ON, Canada). The solvent system used to separate the complex lipid mixture on the C30 column was as follows: Solvent A consisted of acetonitrile: H_2_O (60:40 *v/v*) containing 10 mM ammonium formate and 0.1% formic acid. Solvent B consisted of isopropanol:acetonitrile:water (90:10:1 *v/v/v*) with 10 mM ammonium formate and 0.1% formic acid. The UHPLC-C30RP separation was carried out at 30 °C (column oven temperature) with a flow rate of 0.2 mL/min, and 10 µL of the complex lipid mixture suspended in chloroform:methanol (2:1 *v/v*) was injected into the machine. The following system gradient was used for separating the lipid classes and molecular species: 30% solvent B for 3 min, solvent B increases to 43% over 5 min, then increasing to 50% B in 1 min and to 90% B in over 9 min, and from 90% to 99% B over 8 min, and finally kept at 99% B for 4 min. The column was re-equilibrated to starting conditions (70% solvent A) for 5 min prior to each new injection. Lipid analyses were carried out using a Q-Exactive Orbitrap high-resolution accurate mass tandem mass spectrometer (Thermo-Scientific, Berkeley, CA, USA) coupled with an automated Dionex Ulti-Mate 3000 UHPLC system controlled by Chromeleon software. Full-scan HESI-MS and MS/MS acquisitions were performed on the Q-Exactive Orbitrap mass spectrometer in positive mode. The following parameters were used for the Orbitrap mass spectrometer—sheath gas: 40, auxiliary gas: 2, ion spray voltage: 3.2 kV, capillary temperature: 300 °C; S-lens RF: 30 V; mass range: 200–2000 *m/z*; full-scan mode at a resolution of 70,000 *m/z*; top-20 data dependent MS/MS at a resolution of 35,000 *m/z* and collision energy of 35 (arbitrary unit); injection time 35 min; isolation window: 1 *m/z*; automatic gain control target: 5e5. The instrument was externally calibrated to 1 ppm using ESI negative and positive calibration solutions (Thermo Scientific, Berkeley CA, USA). Tune parameters were optimized using mixtures of lipid standards (Avanti Polar Lipids, Alabaster, AL, USA) in both negative and positive ion modes. LipidSearch version 4.1 (Mitsui Knowledge Industry, Tokyo, Japan) was used for the identification and semi-quantification of the lipid classes and lipid molecular species present in the soaps as unsaponified neutral lipids (TG, MG, DG). The parameters used in LipidSearch for identification (processing) were as follow: target database: Q-Exactive; precursor tolerance: 5 ppm; product tolerance: 5 ppm; product ion threshold: 5%; m-score threshold: 2; Quan *m/z* tolerance: ±5 ppm; Quan RT (retention time) range: ±1 min; use of all isomer filter; ID quality filters A, B, and C; adduct ions: [M+NH_4_]^+^ for positive ion mode. The lipid classes selected for the search were: MG (monoacylglycerol), DG (diacylglycerol), and TG (triacylglycerol). Following identification, the observed lipid classes and molecular species were aligned and merged using the alignment parameters published in our previous work [26].

### 2.4. Microbial Tests

The soap samples were incubated at room temperature for 6 months to determine whether microbial growth would be present over this time (phenotypic microbial identification). Microbial growth was assessed after 6 months using the Lotion Crafter microbial test kit for bacteria, yeast, and mold. The biopaddle slides were gently scrubbed with the surface of the soaps while both sides were coated with the samples. The test strips were incubated using an incubator, and the growth of bacteria was checked within 48 h while yeast and mold were checked within 72 h. Microbial results were determined using evaluation charts showing colonies formed on the slides, which correspond to different degree of microbial contamination (www.lotioncrafter.com/microbial-test-kit-lotioncrafter.html).

### 2.5. Statistical Analysis

The chemical parameters measured including, lipid analysis, phenolic content, antioxidant activity, and oxidation status, were made in four replications. The effects of treatment on the chemical parameters were determined by analysis of variance (ANOVA). Fisher’s LSD test with α = 0.05 was used to compare the means when treatment effects were significant [27]. The compositional change of unsaponified lipids is shown as pie charts. XLSTATS (Premium Version, Addinsoft, Paris, France) was used for the statistical analysis of all other data.

## 3. Results and Discussion

### 3.1. Phenolic Content, Oxidantion Status, and Antioxidant Activity of Natural Herbal Soaps

**Figure 1:**
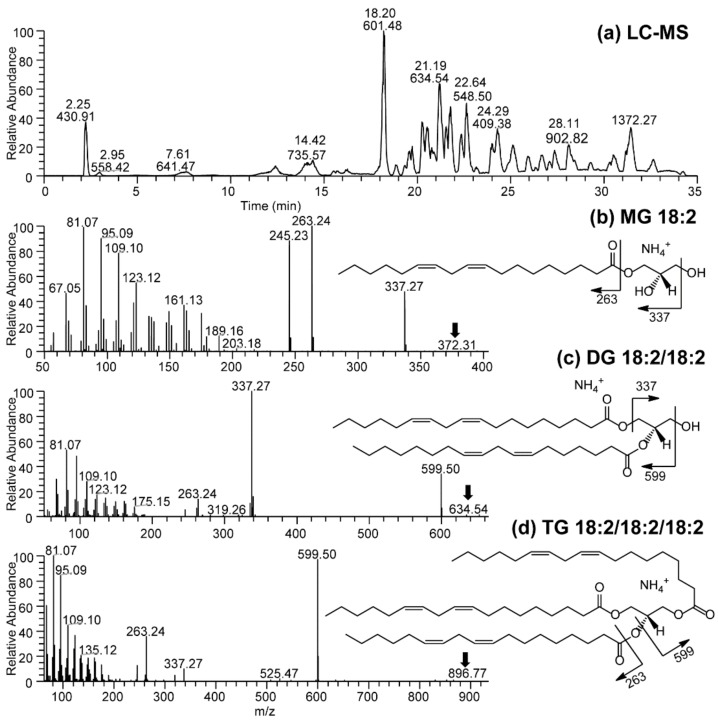
(**a**) UHPLC-MS chromatography and tandem mass spectra (**b**–**d**) of unsaponified monoacylglycerol (MG), diacylglycerol (DG), and triacylglycerol (TG) in base bar soaps, respectively.

**Figure 2:**
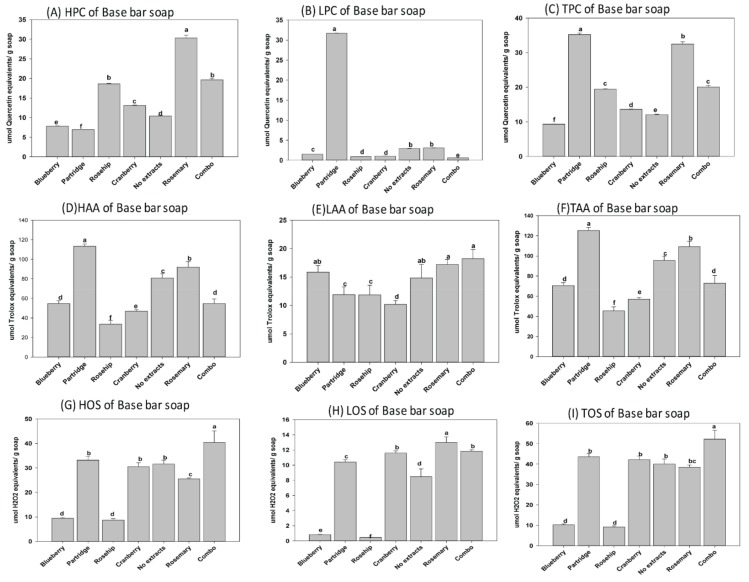
Hydrophilic, lipophilic and total (**A**–**C**) phenolics, (**D**–**F**) antioxidants, and (**G**–**I**) oxidant status in base bar (BB) natural soaps following manufacturing by cold saponification. Bars represent means ± standard errors. Error bars with different letters (a–f) are significantly different among treatments at LSD = 0.05, *n* = 4 per experimental replicate. HPC = hydrophilic phenolic content, LPC = lipophilic phenolic content, TPC = total phenolic content, HOS = hydrophilic oxidant status, LOS = lipophilic oxidant status, TOS = total oxidant status, HAA = hydrophilic antioxidant activity, LAA = lipophilic antioxidant activity, and TAA = total antioxidant activity. Combo = 25% of each of the blueberry, cranberry, and partridge berry and rosehip extracts.

**Figure 3:**
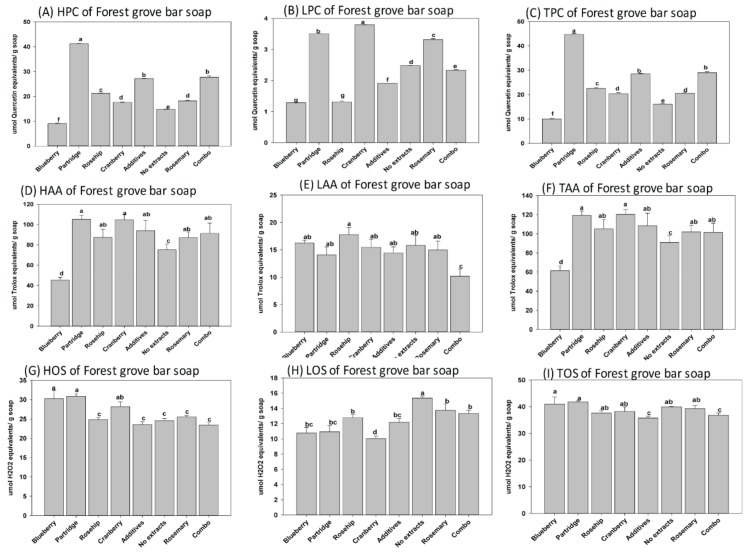
Hydrophilic, lipophilic, and total (**A**–**C**) phenolics, (**D**–**F**) antioxidants, and (**G**–**I**) oxidant status in the forest grove (FG) natural soaps following manufacturing by cold saponification. Bars represent the mean ± standard errors. Error bars with different letters (a–g) are significantly different among treatments at LSD = 0.05, *n* = 4 per experimental replicate. HPC = hydrophilic phenolic content, LPC = lipophilic phenolic content, TPC = total phenolic content, HOS = hydrophilic oxidant status, LOS = lipophilic oxidant status, TOS = total oxidant status, HAA = hydrophilic antioxidant activity, LAA = lipophilic antioxidant activity, and TAA = total antioxidant activity. Combo = 25% of each of the blueberry, cranberry, and partridge berry and rosehip extracts.

**Figure 4:**
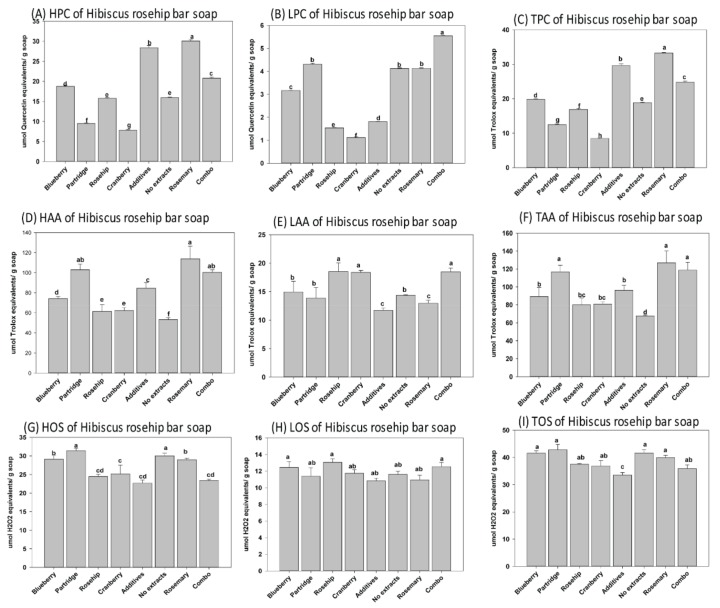
Hydrophilic, lipophilic, and total (**A**–**C**) phenolics, (**D**–**F**) antioxidants, and (**G**–**I**) oxidant status in the hibiscus rosehip (HR) natural soaps following manufacturing by cold saponification. Bars represent the mean ± standard errors. Error bars with different letters (a–g) are significantly different among treatments at LSD = 0.05, *n* = 4 per experimental replicate. HPC = hydrophilic phenolic content, LPC = lipophilic phenolic content, TPC = total phenolic content, HOS = hydrophilic oxidant status, LOS = lipophilic oxidant status, TOS = total oxidant status, HAA = hydrophilic antioxidant activity, LAA = lipophilic antioxidant activity, and TAA = total antioxidant activity. Combo = 25% of each of the blueberry, cranberry, and partridge berry and rosehip extracts.

**Figure 5:**
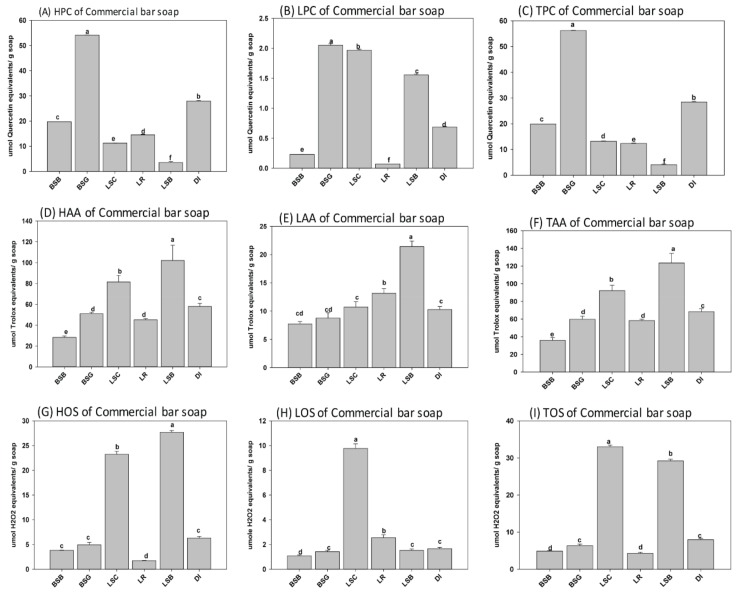
Hydrophilic, lipophilic, and total (**A**–**C**) phenolics, (**D**–**F**) antioxidants, and (**G**–**I**) oxidant status in commercial soaps. Bars represent the mean ± standard errors. Error bars with different letters (a–f) are significantly different among treatments at LSD = 0.05, *n* = 4 per experimental replicate. HPC = hydrophilic phenolic content, LPC = lipophilic phenolic content, TPC = total phenolic content, HOS = hydrophilic oxidant status, LOS = lipophilic oxidant status, TOS = total oxidant status, HAA = hydrophilic antioxidant activity, LAA = lipophilic antioxidant activity, and TAA = total antioxidant activity. Combo = 25% of each of the blueberry, cranberry, and partridge berry and rosehip extracts.

In this study, three natural soaps (i.e., base or control bar (BB), forest grove (FG), and hibiscus rosehip (HR)) produced using cold saponification were selected for evaluations based on popularity among consumers. The base bar (control), containing all the oil blends used as base material in the different soap types, was manufactured specifically for the comparative analysis of each soap. We observed that MG, DG, and TG were present as unsaponified lipids in each soap after cold saponification (Figure 1). Similarly, the phenolics and antioxidants present in the feedstock and extracts were retained in all soaps after cold saponification (Figure 2, Figure 3, Figure 4 and Figure 5). Hydrophilic compounds (phenolic, oxidants, or antioxidants) are soluble in water or aqueous solvents. Conversely, lipophilic compounds are soluble in organic or non-aqueous solvents [9]. In the base bar (BB), the highest phenolic level was observed in samples infused with partridge berry (35.22 µmole quercetin equivalent/g soap) and rosemary (32.49 µmole quercetin equivalent/g soap), followed by combo (combination of berries) and rosehip extracts and then cranberry, while the lowest phenolics were observed in the samples containing no extract (control) or infused with blueberry extract (Figure 2A–C). Concomitant with the high phenolic content, similarly high levels of antioxidant activities were observed in the BB samples containing partridge berry (125.20 µmole Trolox equivalent/g soap) and rosemary extracts (109.22 µmole Trolox equivalent/g soap) (Figure 2D–F). The samples containing no extracts had an intermediate level of antioxidant activities followed by similar levels in samples containing blueberry and a combination of all four wild berry extracts (i.e., cranberry, blueberry, rosehip, and partridge berry). Samples infused with rosehip and cranberry extracts had the lowest antioxidant activity (Figure 2D–F). These results indicate that the cold saponification process used for this natural herbal soap production was suitable to retain high levels of natural antioxidants in the finished soap products. In forest grove (FG), equally high levels of phenolics were observed in samples containing partridge berry extracts (44.72 µmole quercetin equivalent/g soap), followed by the samples containing a combination of all four berry extracts (28.97 µmole quercetin equivalent/g soap) (Figure 3A–C). The FG samples containing blueberry extracts contained the lowest phenolic content (Figure 3A–C). A similar trend in antioxidant activity to that of the phenolic content was observed for FG natural soap samples (Figure 3D–F). The antioxidant levels were similar between samples containing cranberry, partridge berry, additives, rosehip, and rosemary and a combination of all four wild berry extracts (119.23, 120.28, 108.43, 105.18, 102.05, and 101.50 µmole Trolox equivalent/g soap), respectively. The lowest level was recorded in samples containing no extract and wild blueberry extracts (90.93 and 61.49 µmole Trolox equivalent/g soap), respectively. In hibiscus rosehip (HR), the highest level of phenolics was observed in samples containing rosemary extract (33.26 µmole quercetin equivalent/g soap), followed by additives (29.58 µmole quercetin equivalent/g soap), combo (24.78 µmole quercetin equivalent/g soap), blueberry (19.81 µmole quercetin equivalent/g soap), no extract (18.86 µmole quercetin equivalent/g soap), and rosehip (16.86 µmole quercetin equivalent/g soap), while the lowest was observed in HR soaps infused with cranberry extracts (Figure 4A–C). Samples containing rosemary, a combination of all four wild berry extracts, and partridge berry (126.94, 118.88, and 116.83 µmole Trolox equivalent/g soap) had the highest antioxidant levels, respectively, in HR soaps, while the lowest antioxidant activity was observed in HR samples containing no extract (Figure 4D–F).

The level of antioxidants was similar but lower in samples containing blueberry, rosehip, and no berry extracts compared to samples treated with the other wild berry extracts and additives. Wild blueberries are known to have high levels of antioxidants [28]. One surprising discovery from this study is that when natural soaps were infused with NL wild blueberry extracts, the finished soaps retained the lowest levels of antioxidants of the four wild berries used after manufacturing by cold saponification (Figure 2). It appears that the antioxidants and phenolics present in wild blueberries may be more susceptible to degradation during the harsh reaction conditions present during saponification. Furthermore, we observed that partridge berry extracts consistently resulted in high levels of phenolics and antioxidants in natural herbal soaps manufactured using cold saponification. Like wild blueberries, partridge berries are known to contain high levels of polyphenols and antioxidants [28]; these were retained following the cold saponification, suggesting these compounds are more resistant to degradation during natural soap production using this process. Nonetheless, we observed that generally, cold saponification was suitable to retain high levels of natural antioxidants and polyphenols in natural herbal soaps manufactured using wild berry extracts and other natural products in the feedstock. We analyzed the phenolics and antioxidants present in several commercial soaps to get a better understanding of the antioxidant and phenolic contents present in these soaps and to determine how they may be related to the antimicrobial activity compared to natural soaps infused with NL wild berry extracts. In commercial soaps, the highest level of phenolics was observed in BSG (56.24 µmole quercetin equivalent/g soap), followed by DI (28.47 µmole quercetin equivalent/g soap) and BSB (19.83 µmole quercetin equivalent/g soap). Lower levels were observed in LSC and LR (13.19 and 12.37 µmole quercetin equivalent/g soap), respectively, while the lowest was observed in LSB with 4.07 µmole quercetin equivalent/g soap (Figure 5A–C). Conversely, the antioxidant activities of BSG, DI, BSB, LSC, and LR were (59.68, 68.32, 36.02, 92.25, and 58.28 µmole Trolox equivalent/g soap), respectively, were very low compared to their level of phenolics. A similar trend was observed in LSB which had the lowest phenolic content but a high level of antioxidant (123.50 µmole Trolox equivalent/g soap). These results showed that most of the antioxidants activities from commercial brands might not be from polyphenols. In cold saponification, the heat energy release from the reaction is used to facilitate the complete combination of the acids and base (reactants) to form the metal salts produced as the soap product and glycerol as a byproduct [29].

The cold saponification method is preferred by many hand-made soap producers because it is very efficient in retaining the polyphenols, antioxidants, fragrance, and color present in the ingredients used to formulate natural herbal soaps [13], consistent with the findings observed in this study. The oils used as feedstock for manufacturing the natural soaps in this study contained significant levels of unsaturated lipids [3]. The oxidation status after cold saponification and curing of natural herbal soaps were assessed, since unsaturated lipids are known to be susceptible to lipid oxidation [11,22,23]. Significant oxidation occurred in the natural soaps examined in this study (Figure 2, Figure 3 and Figure 4 (G–I)). The oxidation status was higher in BB containing a combination of extract from all four wild berries (52.27 µmole H_2_O_2_ equivalents/g soap). The levels were similar between samples containing partridgeberry, cranberry, no extracts and rosemary (43.58, 42.16, 40.02 and 38.51 µmole H_2_O_2_ equivalents/g soap). Interestingly, samples containing blueberry (10.26 µmole H_2_O_2_ equivalents/g soap) and rosehip (9.15 µmole H_2_O_2_ equivalents/g soap) extracts, which incidentally had the lowest phenolic content, also had the lowest oxidation status. Perhaps the low oxidation status in BB samples containing blueberry and rosehip extracts suggests that the phenolics may be used up due to the fact of protecting the unsaturated fatty acids in the samples from oxidation during saponification. Interestingly, the oxidation status was completely different from the BB samples, when FG soaps were infused with wild berry extracts (Figure 3G–I).

**Figure 6:**
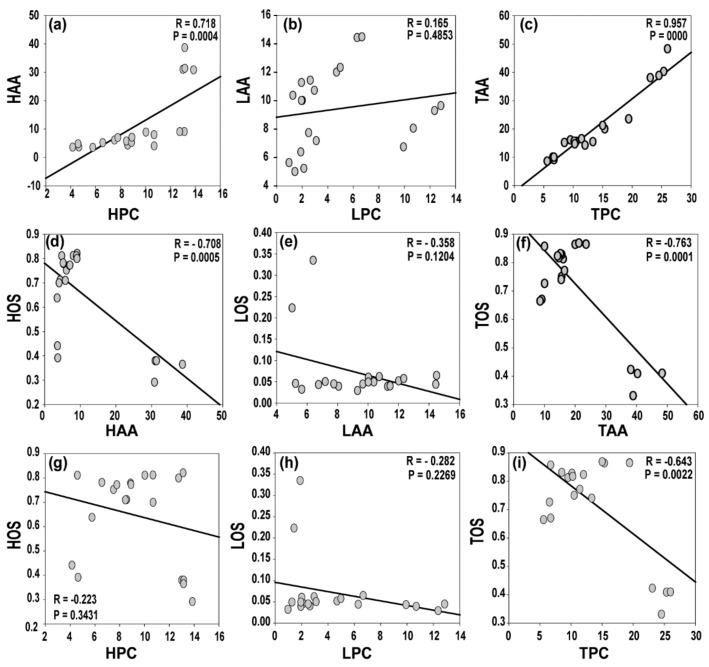
Scatter plots showing relationships between the phenolic content and antioxidant activities in natural soaps formulated with wild berry extracts following cold saponification (**a**–**c**). Scatter plots showing relationships between the antioxidant activities and oxidation status in natural soaps (**d**–**f**). Scatter plots showing relationships between the phenolic content and oxidation status in natural soaps formulated with berries following cold saponification (**g**–**i**). HPC = hydrophilic phenolic content, LPC = lipophilic phenolic content, TPC = total phenolic content, HAA = hydrophilic antioxidant activity, LAA = lipophilic antioxidant activity, TAA = total antioxidant activity, HOS = hydrophilic oxidant status, LOS = lipophilic oxidant status, TOS = total oxidant status.

Surprisingly, the oxidation status was similar among samples regardless of the extracts or additive used in the formulation and production of the FG natural soaps (Figure 3G–I). The HR samples infused with rosehip, cranberry, additives, and a combination of berry extracts contained the lowest oxidation status (37.52, 36.87, 33.50, and 35.92 µmole H_2_O_2_ equivalents/g soap; Figure 4G–I), respectively. This observed oxidation status appears to be proportional to the phenolic content reported in soap samples. Furthermore, and as expected, we observed highly significant correlations between the phenolics and antioxidants present in the natural soaps (Figure 6a–c). Polyphenols are known to contain high levels of antioxidant activity [22,27,30]. These findings suggest polyphenolic compounds contributed significantly to the total antioxidant activities of the examined natural soaps (Figure 6a–c), consistent with other reports in the literature [25,31]. The total phenolic content was observed to be correlated with total antioxidant activity and inversely correlated with total oxidation status (Figure 6d–i), suggesting both the polyphenols and antioxidants retained in the natural herbal soaps were associated with suppressed oxidation [23,32]. There is limited information in the literature, to the best of our knowledge) reporting this relationship in natural herbal soaps manufactured following cold saponification. This may be useful information to artisanal soap makers during their selection of natural berries or fruits to use as additives in their formulation.

### 3.2. Effects of Cold Saponification and Wild Berry Extracts on the Unsaponified Neutral Lipids Composition of Natural Herbal Soaps

**Figure 7:**
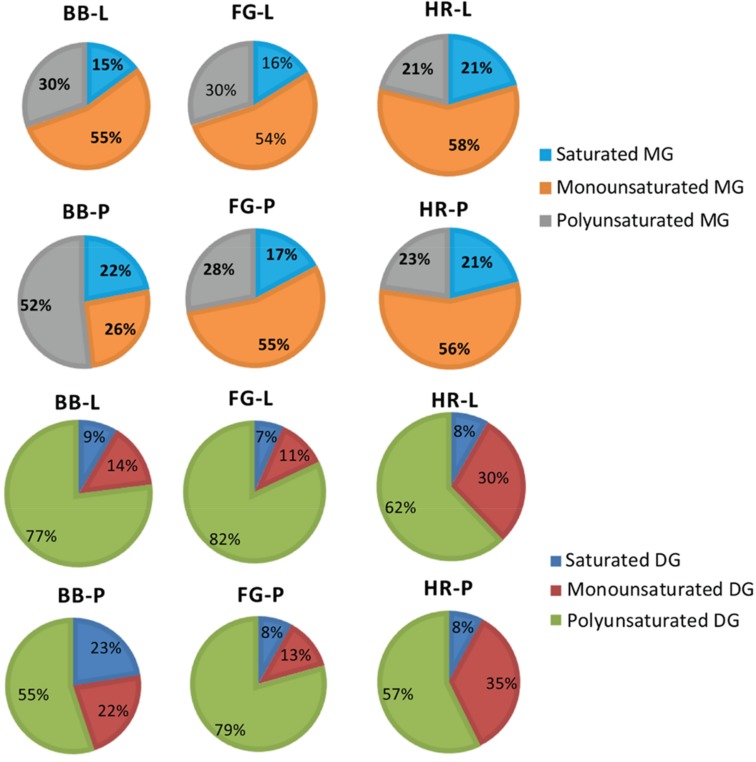
Compositional change of unsaponified monoacylglycerols (MGs) and diacylglycerols (DGs) in natural herbal soaps infused with wild berry extracts. Natural soap acronyms: BB = base bar, FG = forest grove, HR = hibiscus rosehip, L = blueberry, P = partridge berry.

**Figure 8:**
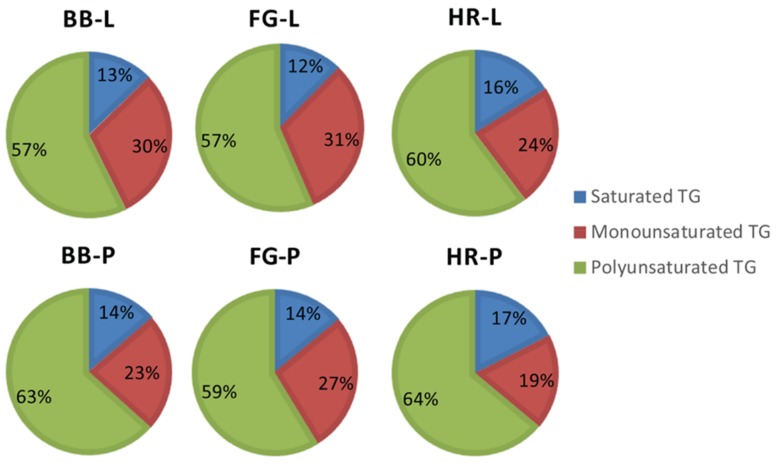
Compositional change in unsaponified triacylglycerols (TGs) in natural herbal soaps infused with wild berry extracts. Natural soap acronyms: BB = base bar, FG = forest grove, HR = hibiscus rosehip, L = blueberry, P = partridge berry.

The texture of the soap was majorly determined by the saturated and unsaturated lipid composition of the product. For instance, saturated lipids confer hardness to the soap while shorter chain length saturated lipids increase solubility of the soap in water as well as cleaning ability. Too much saturated fatty acids can dehydrate skin after use. The unsaturated lipids, on the other hand, act as emollients or moisturizers in soaps. Therefore, mixture of saturated and unsaturated fatty acids in the soap determine the characteristic properties such as hardness, cleansing, ladder, and moisturizing abilities. It was observed that both saturated and unsaturated neutral lipids remained unsaponified in the manufactured natural soaps regardless of feedstock used. The results showed that cold saponification retains a minority of saturated neutral lipids (MGs, DGs, and TGs) in the natural soaps produced ranging from 15–22%, 7–23%, and 12–17%, respectively (Figure 7 and Figure 8).

In contrast to saturated lipids, the majority of the unsaturated neutral lipids (MGs, DGs, and TGs) remained unsaponified in the manufactured natural soaps. These ranged from 26–58% for monounsaturated MGs, 11–35% for monounsaturated DGs, 19–31% for monounsaturated TGs, 26–58% for polyunsaturated MGs, 55–82% for polyunsaturated DGs, and 57–64% for polyunsaturated TGs in all the natural soap produced following cold saponification (Figure 7 and Figure 8). However, the majority of the unsaponified lipids from the manufactured natural soaps were polyunsaturated DGs and TGs (55–82%). We anticipated seeing greater losses in the unsaturated lipids during saponification. The opposite was observed in this study where reduced percent losses as well as increases in the content of unsaturated lipids were observed in the three natural soaps following cold saponification. These findings indicate that considerations should be taken by natural soap manufacturers regarding the effects of cold saponification and feedstock formulation on the texture, ladder, cleansing, and moisturizing abilities of natural soaps, because cold saponification and feedstock appear to significantly modulate the levels of unsaponified saturated and unsaturated neutral lipids (MGs, DGs, and TGs) in the different soap products, and this modulation can vary significantly among soap types based on wild berry or plant extract used. Moreover, polyunsaturated lipids present in MGs, DGs, and TGs are susceptible to oxidation which leads to a reduction in the shelf life of the product (new oxidation compounds generated with rancid aroma tones for example), while the antioxidants are utilized to inhibit the process of oxidation [33].

### 3.3. Effects of Antioxidants on Shelf Life of the Natural Herbal Soaps

The presence of microbial growth on the samples stored at room temperature over a 6 month test period was evaluated using the Lotioncrafter Microbial Test Kit (Cottonwood AZ, USA) (Table 1). Samples were also evaluated weekly to determine any appearance of off color/odor and visual appearance of microbial growth. No off color, odor, or visual appearance of microbial growth was observed on the test samples over the test period. However, when samples were tested to evaluate the presence of microbial growth using Lotioncrafter Microbial Test Kits, after 6 months, the appearance of microbial growth was observed on all four types of natural soaps evaluated (Table 1). All the commercial brands tested positive for microbial growth at first testing. However, it is important to point out that the commercial brands were purchased from stores, and we have no indication of how long they were sitting on the shelf (Table 1). The results indicate that at least a 6 month shelf life was achieved for the tested natural soaps using microbial growth as a proxy for shelf life. The results also indicated that addition of some of the natural berry extracts appears to promote microbial growth, while others were inhibitory in the different natural soap formulations.

In BB natural soap, the addition of Rosemary extracts (M) and blueberry and cranberry (C) promoted microbial growth. Conversely, partridge berry, rosehip, and the combination extracts inhibited microbial growth, indicating a shelf life longer than 6 months for the base bar is possible by adding partridge berry, rosehip, a combination extract, or no extract at all (Table 1). In the FG natural soaps, the addition of rosemary and the combination extracts were effective in inhibiting microbial growth and could be used to possibly extend the shelf life beyond 6 months in the forest grove soaps (Table 1). In the HR, only the rosemary extract was effective in inhibiting microbial growth at 6 months, indicating the potential application of rosemary extract in extending the shelf life of hibiscus rosehip soap bars beyond 6 months (Table 1). Polyphenols in fruits extracts, as well as those from herbs such as rosemary have been reported to contain antimicrobial properties [34,35]. It appears that the varied level of polyphenols in the different plant extracts contributed to the differential microbial growth observed when natural soaps were infused with wild berry extracts. In summary, the data revealed that attention should be paid to the choice of wild berry extract to be used in different natural soap formulations, because some extracts were inhibitory (potentially extending shelf life) while others promoted microbial growth (potentially reducing the shelf life) in the end products (Table 1). In addition, berry extracts are found to contain high levels of phenolic compounds which are demonstrated to have antimicrobial activity in in vitro and in vivo studies [16,17]. Furthermore, the precise antimicrobial activities of phenolics in berries mostly depend on factors such as the phenolic class, their concentration level, and type [28]. Also, the numbers under each figure (evaluation charts) give an estimate of the total number of colony-forming units present in the sample. In this paper, we used the absence or presence of visual microbial infection on the media as a proxy for shelf life rather than give an estimate of the number of colony-forming units present in each treatment.

## 4. Conclusions

Currently, to the best of our knowledge, there is limited information in the scientific literature related to how variation in plant extracts and additives could modulate the unsaponified neutral lipids, antioxidants, phenolics content, shelf life, and perceptual quality of natural herbal soaps. The results obtained from this study seek to fill this knowledge gap. This study demonstrated that the natural soaps produced with wild berry extracts and natural additives following cold saponification retained significant levels of antioxidants, phenolics, and unsaponified neutral lipids (DG, MG, and TG) in the final products. Most of the unsaturated lipids remained unsaponified after soap production via cold saponification. Furthermore, the phenolics and antioxidants contents were inversely correlated with the oxidation status of the natural herbal soaps evaluated in this study. The antioxidant and phenolics varied significantly among different types of soap even though the same wild berry extracts and additives were used. As such, keen attention should be paid to the choice of wild berry extract to be used in different natural soap formulations, because some extracts were inhibitory (potentially extending shelf life) while others promoted microbial growth (potentially reducing the shelf life) in the end products. Additionally, the total phenolic content (TPC) and total antioxidant activities (TAA) of the natural herbal soaps were relatively higher compared with the commercial types, leading to a potential improvement in shelf life, although we are not sure how long the commercial soaps were on the store shelf prior to purchase for this study. Nonetheless, these findings are very significant as a source of information to guide artisanal natural soap production, which is very scarce in the scientific literature. We believe that the information presented here will encourage further studies by other researchers in the scientific community to further enhance the knowledge that may be of great value to the growing specialty of the hand-made soap industry.

## Figures and Tables

**Table 1:**
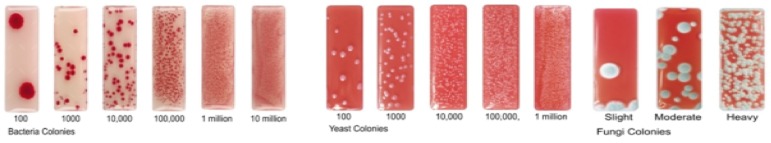
Microbial growth in different natural and commercial soaps. (+) = indication of microbial growth, (−) = indication of microbial inhibition. Natural soap acronyms: BB = base bar, FG = forest grove, HR = hibiscus rosehip, A = essential old blend additives, L = blueberry, P = partridge berry, S = rosehip, C = cranberry, M = rosemary extracts, Combo = combination of berry extracts. Picture represents Lotioncrafter panel indicating colonies of bacteria, yeast, and fungi in the test samples.

Sample	Bacteria	Yeast	Molds
Base Bar - L	−	−	+
Base Bar - P	−	−	−
Base Bar - S	−	−	−
Base Bar - C	+	−	−
Base Bar - No Extracts	−	−	−
Base Bar - M	+	−	−
Base Bar - Combo	−	−	−
Forest Grove - L	+	+	+
Forest Grove - P	+	−	+
Forest Grove - S	+	+	−
Forest Grove - C	+	+	+
Forest Grove - A	+	−	−
Forest Grove - No Extracts	+	−	−
Forest Grove - M	−	−	−
Forest Grove Combo	−	−	−
Hibiscus Rosehip - L	−	−	+
Hibiscus Rosehip - P	+	−	−
Hibiscus Rosehip - S	+	−	−
Hibiscus Rosehip - C	+	+	−
Hibiscus Rosehip - A	−	+	−
Hibiscus Rosehip -No Extracts	+	−	+
Hibiscus Rosehip - M	−	−	−
Hibiscus Rosehip - Combo	+	−	−
Commercial - BSB	+	+	+
Commercial - BSG	+	+	+
Commercial - LSC	+	−	+
Commercial - LR	+	+	+
Commercial - LSB	+	+	−
Commercial - DI	+	−	−
